# Supplementary material for: Reaction Time and Visual Memory in Connection with Alcohol Use in Schizophrenia and Schizoaffective Disorder
Source: Brain Sci. 2021 May 23;11(6):688. doi: 10.3390/brainsci11060688 (PMC8224767; doi:10.3390/brainsci11060688)
Supplement: Supplementary file 1 [file brainsci-11-00688-s001.zip › brainsci-1204494-supplementary.pdf]

brain sciences

MDPI

Supplementary Table S1. Association between background factors and hazardous drinking in schizophrenia and schizoaffective disorder.

		**Male**			Female		
		Hazardous drinking			Hazardous drinking		
		No	Yes	*p*	No	Yes	*p*
n		1276	435		1390	261	
Age (mean (SD))		45.79 (13.03)	40.84 (11.54)	<0.001	47.45 (13.11)	40.19 (11.97)	<0.001
Education (N (%))	No matriculation examination	889 (69.7)	302 (69.4)		818 (58.8)	163 (62.5)	0.308
	Matriculation examination	387 (30.3)	133 (30.6)	0.971	572 (41.2)	98 (37.5)	
Age at time of first psychotic episode (mean (SD))		26.77 (8.48)	27.04 (7.57)	0.561	28.36 (9.71)	26.14 (8.13)	0.001
Household patterns (N (%))	With spouse	152 (11.9)	48 (11.0)	0.685	362 (26.0)	79 (30.3)	0.180
	Other	1124 (88.1)	387 (89.0)		1028 (74.0)	182 (69.7)	
Psychotropic medication (N (%))	No	35 (2.7)	7 (1.6)		30 (2.2)	3 (1.1)	
	Yes	1240 (97.2)	428 (98.4)		1356 (97.6)	258 (98.9)	
	Missing	1 (0.1)	0 (0.0)	0.352	4 (0.3)	0 (0.0)	0.385

Supplementary Table S2. Distribution of RT median, RT SD, PAL first trial memory scores (FTMS), and PAL total error adjusted in the study population.

	**Min**	**1st Qu.**	**Median**	**Mean**	**3rd Qu.**	**Max**
RT Median	288	298	434.5	450.2	482	1610
RT SD	14.78	37.98	49.95	66.50	69.87	922.02
PAL FTMS	0.0	4.0	8.0	8.3	12	20
PAL total error adjusted	0.0	14	35	33.16	50	70

Supplementary Table S3. RT median and RT SD *p*-values for background factors and alcohol use disorder in schizophrenia and schizoaffective disorder.

***p*-Values**					
	**Male**		**Female**		
	RT		RT		
	Median	SD	Median	SD	Test
Age	<0.001	<0.001	<0.001	<0.001	Spearman
Education	0.563	0.104	<0.001	<0.001	Spearman
Age at time of first psychotic episode	<0.001	<0.001	<0.001	<0.001	Spearman
Alcohol use disorder	0.445	0.245	0.178	0.211	Point biserial

Supplementary Table S4. PAL first trial memory scores (FTMS) for background factors and alcohol use patterns in schizophrenia and schizoaffective disorder.

		**Male**			**Female**		
		PAL FTMS			PAL FTMS		
		0	1	*p*	0	1	*p*
n		1462	79		1361	100	
Age (mean (SD))		44.86 (12.58)	33.77 (9.41)	<0.001	46.28 (12.97)	37.44 (10.87)	<0.001
Education (N (%))	Matriculation examination	452 (30.9)	35 (44.3)	0.018	564 (41.4)	58 (58.0)	0.002
	No matriculation examination	1010 (69.1)	44 (55.7)		797 (58.6)	42 (42.0)	
Age at time of first psychotic episode (mean (SD))		26.90 (8.26)	24.38 (5.93)	0.008	28.11 (9.69)	25.93 (7.69)	0.028
Household patterns (N (%))	With spouse	158 (10.8)	16 (20.3)	0.016	362 (26.6)	34 (34.0)	0.136
	Other	1304 (89.2)	63 (79.7)		999 (73.4)	66 (66.0)	
Psychotropic medication (N (%))	No	34 (2.3)	7 (8.9)		26 (1.9)	5 (5.0)	
	Yes	1427 (97.6)	72 (91.1)		1334 (98.0)	94 (94.0)	
	Missing	1 (0.1)	0 (0.0)	0.002	1 (0.1)	1 (1.0)	0.006
Hazardous drinking (N (%))	No	1101 (75.3)	51 (64.6)	0.044	1146 (84.2)	80 (80.0)	0.335
	Yes	361 (24.7)	28 (35.4)		215 (15.8)	20 (20.0)	
Alcohol use disorder (N (%))	No	1037 (70.9)	62 (78.5)	0.188	1143 (84.0)	90 (90.0)	0.145
	Yes	425 (29.1)	17 (21.5)		218 (16.0)	10 (10.0)	

Supplementary Table S5. PAL total error adjusted scores for background factors and alcohol use patterns in schizophrenia and schizoaffective disorder.

		**Male**			**Female**		
		PAL total error adjusted			PAL total error adjusted		
		0	1		0	1	
n		241	1300		297	1164	
Age		35.46 (10.11)	45.93 (12.43)	<0.001	37.82 (10.90)	47.68 (12.76)	<0.001
Education	No matriculation examination	129 (53.5)	925 (71.2)		129 (43.4)	710 (61.0)	
	Matriculation examination	112 (46.5)	375 (28.8)	<0.001	168 (56.6)	454 (39.0)	<0.001
Age at time of first psychotic episode		24.34 (5.99)	27.23 (8.45)	<0.001	25.94 (7.70)	28.47 (9.94)	<0.001
Household pattern	Alone	174 (72.2)	1038 (79.8)	0.031	167 (56.2)	770 (66.2)	<0.001

	With children without spouse	1 (0.4)	5 (0.4)		23 (7.7)	32 (2.7)	
	With parents or siblings	30 (12.4)	119 (9.2)		19 (6.4)	54 (4.6)	
	With spouse	20 (8.3)	97 (7.5)		56 (18.9)	241 (20.7)	
	With spouse and children	16 (6.6)	41 (3.2)		32 (10.8)	67 (5.8)	
Psychotropic medication	No	11 (4.6)	30 (2.3)		10 (3.4)	21 (1.8)	
	Yes	230 (95.4)	1269 (97.6)		286 (96.3)	1142 (98.1)	
	Missing	0 (0.0)	1 (0.1)	0.124	1 (0.3)	1 (0.1)	0.143
Hazardous drinking	No	166 (68.9)	986 (75.8)	0.027	239 (80.5)	987 (84.8)	0.085
	Yes	75 (31.1)	314 (24.2)		58 (19.5)	177 (15.2)	
Binge drinking	Never	100 (41.5)	714 (54.9)		170 (57.2)	820 (70.4)	
	Monthly or less frequently	106 (44.0)	430 (33.1)	0.001	111 (37.4)	285 (24.5)	<0.001
	Weekly or more frequently	35 (14.5)	156 (12.0)		16 (5.4)	59 (5.1)	
Alcohol use disorder	No	186 (77.2)	913 (70.2)	0.035	259 (87.2)	974 (83.7)	0.160
	Yes	55 (22.8)	387 (29.8)		38 (12.8)	190 (16.3)	

Supplementary Table S6. RT median and RT SD for hazardous drinking in schizophrenia and schizoaffective disorder.

		**Male**			**Female**		
		Hazardous drinking			Hazardous drinking		
		0	1	*p*	0	1	*p*
n		1276	435		1390	261	
RT	Median (mean (SD))	453 (96.06)	438.30 (79.04)	0.004	455.75 (98.50)	426.66 (64.39)	<0.001
	SD (mean (SD))	67.93 (71.75)	53.65 (32.57)	<0.001	70.60 (75.62)	59.26 (54.18)	0.021

Supplementary Table S7. Cohen's d measure of effect.

	**Male**	**Female**
Hazardous drinking		
RTI SD	0.22 (0.11, 0.33)	0.16 (0.05, 0.27)
RTI Median	0.16 (0.05, 0.27)	0.31 (0.18, 0.44)
PAL FTMS	0.08 (0.03, 0.19)	0.12 (0.03, 0.26)
PAL TEA	0.13 (0.02, 0.25)	0.13 (0.00, 0.27)
Alcohol disorder		
RTI SD	0.02 (0.05, 0.12)	0.02 (0.12, 0.15)
RTI median	0.02 (0.01, 0.10)	0.08 (0.00, 0.19)
PAL FTMS	0.11 (0.25, 0.03)	0.08 (0.03, 0.19)
PAL TEA	0.12 (0.23, 0.01)	0.12 (0.01, 0.26)
